# AtPAP1 Interacts With and Activates SmbHLH51, a Positive Regulator to Phenolic Acids Biosynthesis in *Salvia miltiorrhiza*

**DOI:** 10.3389/fpls.2018.01687

**Published:** 2018-11-20

**Authors:** Yucui Wu, Yuan Zhang, Lin Li, Xiaorong Guo, Bin Wang, Xiaoyan Cao, Zhezhi Wang

**Affiliations:** ^1^National Engineering Laboratory for Resource Development of Endangered Crude Drugs in Northwest of China, Key Laboratory of the Ministry of Education for Medicinal Resources and Natural Pharmaceutical Chemistry, Shaanxi Normal University, Xi’an, China; ^2^School of Landscape and Ecological Engineering, Hebei University of Engineering, Handan, China; ^3^College of Chemistry, Biology and Materials Science, East China University of Technology, Nanchang, China

**Keywords:** AtPAP1, phenolic acids, salvianolic acid B, *Salvia miltiorrhiza*, SmbHLH51

## Abstract

Phenolic acids from *Salvia miltiorrhiza* have drawn considerable attention in recent years because of their remarkable pharmacological activities. We previously reported that *Arabidopsis thaliana* transcription factor production of anthocyanin pigment 1 (AtPAP1) has strong capability to promote the production of phenolic acids in *S. miltiorrhiza*. However, the responsible molecular mechanism is unclear. Here, we analyzed the transcriptome of transgenic *S. miltiorrhiza* that over-expressed *AtPAP1*. Transcriptome analysis revealed 4,152 genes that were differentially expressed due to ectopic *AtPAP1* overexpression. *SmbHLH51*, a novel bHLH gene significantly up-regulated by constitutive expression of *AtPAP1*, was isolated from *S. miltiorrhiza* for detailed functional characterization. SmbHLH51 localizes in the nuclei and interacts with AtPAP1, indicating that they probably comprise a regulatory transcription complex. Enhanced or reduced expression of *SmbHLH51* was achieved in *S. miltiorrhiza* by gain- or loss-of-function assays, respectively, revealing that SmbHLH51 is a positive transcriptional regulator of the pathway for phenolic acid biosynthesis. We propose that applying this functional genomics approach through the transcriptomic analyses is an efficient means for identifying novel genes involved in plant secondary metabolism.

## Introduction

*Salvia miltiorrhiza* Bunge, a well-known member of the Labiatae family, is emerging as a model plant system for research in regulation of secondary metabolites because of its significant medicinal values, high transformation efficiency and published genome data (Shao and Lu, 2013; Xu et al., 2015b). Its dry roots or rhizomes (called ‘danshen’ in Chinese) have been widely and successfully used in traditional Chinese medicine for thousands of years to treat cardiovascular disease, amenorrhea, and dysmenorrhea (Cheng, 2006). The bioactive components of *S. miltiorrhiza* are divided into two groups: lipid-soluble tanshinones and water-soluble phenolic acids. The latter group includes rosmarinic acid (RA) and salvianolic acid B (Sal B), which are attracting increased attention because of their marked pharmacological activities coupled with their traditional use as herbs steeped in boiling water in China (Petersen and Simmonds, 2003; Wu et al., 2012b). Because its active ingredients have such high economic value, studies are now focusing more on the pathway for phenolic acid biosynthesis (Hua et al., 2011; Wang et al., 2015). The phenolic acids distribute in different parts of *Salvia miltiorrhiza* and the content of total phenolic acids from leaves was significantly higher than those in roots and stems (Li et al., 2011).

Generally, overexpression of transcription factor (TF) that regulate the expression of genes involved in entire metabolic pathways is an effective tool for engineering high levels of metabolites (Butelli et al., 2008). Our research group was the first to show that the formation of health-promoting phenolic acids, especially Sal B, in *S. miltiorrhiza*, can be enriched by the ectopic overexpression of the *Arabidopsis thaliana* MYB TF production of anthocyanin pigment 1 (AtPAP1) (Zhang et al., 2010). However, the underlying molecular mechanism is unclear.

The phenylpropanoid pathway in different plant organs and tissues of higher plants is under the control of specific R2R3-MYB TFs and bHLH families (Xue et al., 2011). In *S. miltiorrhiza*, only a few endogenous MYBs have been functionally characterized to be involved in the regulation of phenolic acids biosynthesis (Zhang et al., 2013; Hao et al., 2016; Ding et al., 2017; Li et al., 2018). Among the 127 bHLHs identified in *S. miltiorrhiza* (Zhang et al., 2015), none has been verified to be involved in the regulation of phenolic acids by now. The bHLH genes are regulated by R2R3-MYB TFs. For example, constitutive expression of *AcMYB110* that is highly activated in the anthocyanin pathway in tobacco up-regulates interacting endogenous NtAN1 bHLH (Montefiori et al., 2015). Ectopic expression of the MYB *NtAN2* in tobacco promotes anthocyanin biosynthesis by up-regulating the expression of biosynthetic genes and bHLH *NtAN1* (Bai et al., 2011). Activity of TT8 is strongly affected in AtPAP1 overexpressors (Baudry et al., 2006). Besides, it’s well documented that flavonoid biosynthesis is regulated by MYB-bHLH-WDR (MBW) complexes (Xu et al., 2015a). In *Arabidopsis*, AtPAP1 can interact with TT8, GL3, EGL3, or AtMYC1, together with TTG1 (WDR family), to form MBW complexes and activate flavonoid biosynthesis (Feller et al., 2011).

Recently, transcriptome analysis has been used to identify candidate genes on the biosynthesis pathways of secondary metabolites and explore the potential mechanisms associated with important traits in *S. miltiorrhiza* (Yang et al., 2013; Song et al., 2017; Wei et al., 2017; Pei et al., 2018). Manipulating a single TF can reprogram the transcriptome profiling and is an effective way to regulate some specific secondary metabolites. Since ectopic expression of *AtPAP1* in *S. miltiorrhiza* greatly improved the level of Sal B (Zhang et al., 2010), the expression levels of many genes associated with Sal B biosynthesis pathway must be changed. Comparison of the transcriptomes between transgenic *S. miltiorrhiza* over-expressing *AtPAP1* and the wild-type (WT) plants will help to identify not only novel genes associated with Sal B biosynthesis but also specific targets of AtPAP1, which will improve our insights into the Sal B accumulation mechanisms in *S. miltiorrhiza*.

Here, we report an examination of the transcriptome of *AtPAP1*-overexpressing *S. miltiorrhiza*. Our objectives were to investigate (1) the role of a single TF in global gene expression, and (2) the specific target/mechanism that results in the enrichment of phenolic acid biosynthesis in *S. miltiorrhiza.* To answer the first question, we studied transcriptomics using the Solexa/Illumina digital gene expression (DGE) system. From this we determined that a set of genes involved in phenolics accumulations are up-regulated in conjunction with the production of phenolic acids, and we identified the genes that function in that process. *SmbHLH51*, significantly up-regulated by constitutive expression of *AtPAP1*, was identified as a positive regulator for the phenolic acids biosynthesis by overexpressing and suppressing *SmbHLH51* in *S. miltiorrhiza.* Our results indicate that AtPAP1 stimulates phenolic acid biosynthesis in *S. miltiorrhiza* by activating the expression of *SmbHLH51* and probably forming a potential transcription complex with SmbHLH51.

## Materials and Methods

### Experimental Materials

Plant materials were cultured under the conditions described in our previous study (Hua et al., 2012). Transgenic line AtPAP1-14 (Zhang et al., 2010) and wild-type (WT) plants of *S. miltiorrhiza* were sampled on day 180 (D180) of growth for use in transcriptome and metabolome analysis. All chemicals were purchased from Sigma Chemical Co. (St. Louis, MO, United States). Solvents were HPLC grade. Standards of RA and Sal B were obtained from the National Institute for the Control of Pharmaceutical and Biological Products (Beijing, China). All standards were prepared as stock solutions in methanol and were stored in the dark at -18°C. Primer pairs are listed in Supplementary Table S1.

### Transcriptome Analysis

We performed DGE profile analysis of AtPAP1-14 and WT plants by Solexa sequencing, as described before (Ge et al., 2015). Briefly, all clean tags obtained by filtering out any adaptor-only tags and low-quality tags were annotated based on *S. miltiorrhiza* reference genes (Hua et al., 2011). We identified DEGs based on false discovery rates ≤ 0.001, *P*-values < 0.005, and an estimated absolute log_2_ fold-change of ≥1 in sequence counts across libraries. Significantly enriched metabolic pathways were functionally classified into several categories with Kyoto Encyclopedia of Genes and Genomes (KEGG^1^) by comparing those DEGs with the control genome background. Pathways with Q-values < 0.05 were considered significantly enriched in DEGs.

### Isolation and Sequence Analysis of *SmbHLH51*

We designed primers *bHLH51*-F/*bHLH51*-R to clone *SmbHLH51* (GenBank Accession Number KT215166) with LA DNA polymerase (Takara, Japan) at both the genomic DNA level and the transcriptome cDNA level. Total RNA was isolated from 3-month-old plantlets of *S. miltiorrhiza* with a Plant RNA kit (OMEGA, United States), according to the manufacturer’s protocol, and was converted into cDNA using a primeScript RT reagent kit (TaKaRa, Dalian, China). Sequencing-verified *SmbHLH51* cDNA cloned in pMD19-T (pMD19-*cSmbHLH51*) was used as template in some of our PCR reactions.

Combining the DNA and cDNA sequences, we obtained a diagram of exon/intron structures using public online software from the Gene Structure Display Server^2^. For our homology search we used BLAST^3^. The MW and pI of each corresponding protein were predicted with the ProParam tool^4^. Homologous bHLH protein sequences were aligned by CLUSTAL W (version 1.83) (Thompson et al., 1994) and the phylogenetic tree was constructed with MEGA 6.0 software (Tamura et al., 2013) and the Neighbor-Joining method (Gascuel and Steel, 2006).

### Subcellular Localization Assay of SmbHLH51

The subcellular localization assay was performed by transiently expressing the SmbHLH51-GFP fusion protein in onion epidermal cells. The entire CDS region of *SmbHLH51* was amplified from pMD19-*cSmbHLH51* with primers 207-*SmbHLH51*-F/R, which contain the *att*B1/*att*B2 sites. The PCR products were cloned into entry vector pDONR207, using the BP recombination reaction, and then transferred into destination vector pEarleyGate103 (Earley et al., 2006) through an LR reaction, according to the protocol from the Gateway technology manufacturer (Invitrogen, United States). The pEarleyGate103-*SmbHLH51* vector was transformed into onion epidermal cells by particle bombardment at a helium pressure of 1100 psi with the PDS-1000/He system (Bio-Rad, United States) and observed using a Leica DM6000B microscope (Leica, Germany) as described before (Li et al., 2018). The pEarleyGate103 plasmid was transformed into onion epidermal cells as a positive control.

### Monitoring Transient Expression in Tobacco for Assaying Transcriptional Activation

We used *AtPAP1*-overexpressing plasmid p*AtPAP1*-OE, which we had constructed in our laboratory as the effector plasmid (Zhang et al., 2010). Fragments of the *SmbHLH51* promoters were cloned using primers Pro-SmbHLH51-F/R and fused with the *GUS* reporter gene in pCAMBIA1391Z to obtain the reporter plasmid p*ProbHLH51*-1391Z. The resulting constructs were transformed into the *A. tumefaciens* GV3101 strain. Tobacco plants were grown in a light incubator to the four- to six-leaf stage before being infiltrated with *A. tumefaciens* (Yin et al., 2010). After they were then grown in the dark for 24 h, they were exposed to a 16-h/8-h light/dark cycle for 48 h at 23°C. The infiltrated tobacco leaves were used for GUS staining (Jefferson et al., 1987).

### Yeast One-Hybrid (Y1H) Assays

The ORF of *AtPAP1* (GenBank Accession Number NM_104541.3) was amplified by PCR using primers containing *Bam*HI and *Xho*I restriction sites and then fused to the GAL4 activation domain in vector pGADT7-Rec2 (Clontech) to create the fusion proteins pGADT7-*AtPAP1*. A trimer of MBSII element (AAAAGTTAGTTA) and A trimer mutant sequence (AAAAGTAAGGTA) were synthesized and cloned into pHIS2 (Clontech) to obtain pHIS2-MBSII and pMutant-MBSII, respectively. These recombinant vectors were co-transformed into yeast strain Y187 according to the reported protocol (Huang et al., 2013). The transformed cells were cultured on an SD/-Leu/-Trp medium and then selected on an SD/-Leu/-Trp/-His medium supplemented with 60 mM 3-amino-1,2,4-triazole to examine any protein-DNA interactions.

### Yeast Two-Hybrid (Y2H) Assays

For Y2H assays, the ORFs of *AtPAP1* and *SmbHLH51* were cloned into the entry vector pDONR207 and then, respectively, transferred into Gateway vectors pGADT7 and pGBKT7 according to the manufacturer’s protocol. The resulting pGADT7-*AtPAP1* and pGBKT7-*SmbHLH51* were transformed into yeast strain AH109. Primers 207-*AtPAP1*-F/R and 207-*SmbHLH51*-F/R used for vector construction are listed in Supplementary Table S1. The interaction assays were performed according to the Matchmaker Gold Yeast Two-Hybrid System manufacturer’s protocol (Clontech, United States), and Y2H images were taken on day 5 of incubation.

### Firefly Luciferase Complementation Imaging Assay

*AtPAP1* was fused to the N-terminal domain of firefly luciferase (Nluc) of p1301-NLuc to produce destination vector p*AtPAP1-*NLuc. *SmbHLH51* and *AtTT8* were fused to the C-terminal domain of firefly luciferase (Cluc) of p1301-CLuc to obtain p*SmbHLH51*-CLuc and p*AtTT8*-CLuc. The p*AtPAP1*-NLuc and p*AtTT8*-CLuc served as positive controls. Primers *AtPAP1*-NLF/R, *AtTT8*-CLF/R, and SmbHLH51-CLF/R (Supplementary Table S1) were used for constructing the vectors, which were transformed into the *A. tumefaciens* GV3101 strain. Cultures harboring each of the CLuc and NLuc constructs were co-infiltrated into fully expanded tobacco leaves before Luc activity was visualized and pictured with IVIS Spectrum (Xenogen, United States) (Wu et al., 2012a).

### Construction of Plant Expression Vectors and Plant Transformation

To construct the *SmbHLH51-*overexpressing vectors, we designed gene-specific primers OEbHLH51-F and OEbHLH51-R (Supplementary Table S1) to amplify *SmbHLH51*. The PCR products were digested with *Kpn*I/*Bam*HI (Takara) and ligated into the pKANNIBAL vector (Wesley et al., 2001) and sub-cloned into the pART27 vector to generate overexpression plasmid pAK–*SmbHLH51* as described previously (Wang et al., 2017).

To generate the RNAi plasmid, a 260-bp fragment of *SmbHLH51* cDNA (located from coding sequence positions 23–283 bp) was amplified with primers bHLH37i-F/R (Supplementary Table S1) and used to construct RNAi vector p*SmbHLH51*–RNAi according to previously published protocols (Wang et al., 2017).

Transgenic plants were generated through *Agrobacterium*-mediated transformation (Yan and Wang, 2007). In parallel, plasmid pAK, constructed in our laboratory (Wu et al., 2014), was introduced into WT *S. miltiorrhiza* as the empty-vector control PDK.

### Transformant Selection and Transcript Analysis

To evaluate whether the overexpressing and interfering box had been integrated into the transgenic plant genome, we amplified the 35S promoter from isolated genomic DNA, using previously published protocols (Wu et al., 2014).

Transcript abundances for *SmbHLH51* were analyzed by real-time quantitative PCR (RT-qPCR), using *Actin* as the internal reference gene (Yang et al., 2010). Relative gene expression level was performed using the 2^-ΔΔC(T)^ method (Livak and Schmittgen, 2001). Based on the transcript levels of *SmbHLH51*, two *SmbHLH51*-OE lines (OE1 and OE35) and two *SmbHLH51*-RNAi lines (in4 and in10) were selected to detect the expression levels of 19 genes for enzymes involved in pathways for phenolic acid, flavonoid, and lignin biosynthesis. All experiments were performed on three independent biological samples, with each including three technical replicates. All primers are listed in Supplementary Table S1.

### HPLC Analysis of Phenolic Acids

Roots were collected from 2-month-old *SmbHLH51*-overexpressing, *SmbHLH51*-RNAi and control tube plantlets. Extractions and HPLC analysis of phenolic acids were performed as described before except that the LC-2010A HPLC system was substituted by LC-2010 CHT (Zhang et al., 2010).

### Determination of Total Phenolic, Total Flavonoid, and Anthocyanin Concentrations

The levels of total phenolics, total flavonoids, and anthocyanins in 2-month-old transgenic and control tube plantlets were determined by our laboratory procedures (Zhang et al., 2010).

## Results

### Gene Expression Profile of *AtPAP1*-Overexpressing (AtPAP1-14) and WT *Salvia miltiorrhiza*

**FIGURE 1:**
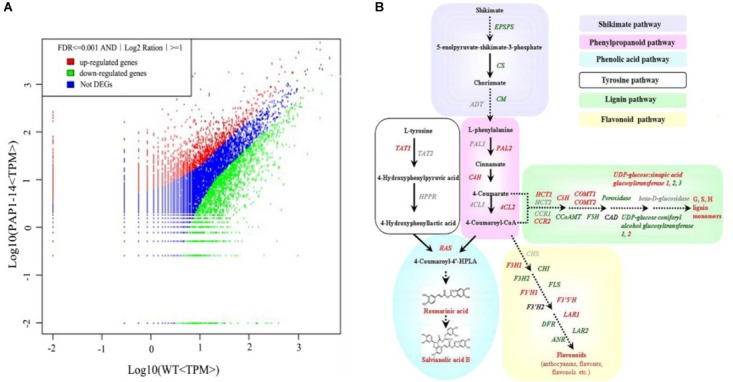
Transcriptome response of *Salvia miltiorrhiza* to overexpression of *AtPAP1*. **(A)** Comparison of gene expression between two libraries, with each normalized to one million tags. Red dots represent transcripts more prevalent in AtPAP1-14 library; green dots, those present at lower frequency in transgenic plants; and blue dots, transcript levels with no significant change. Parameters “FDR < 0.001” and “log_2_Ratio ≥ 1” were used as threshold to judge significance of differences in expression. **(B)** Proposed biosynthetic pathway for phenolics, beginning with core phenylpropanoid and tyrosine pathways, and leading to three major branch pathways: phenolic acid, flavonoid, and lignin (red, upregulated genes; green, downregulated). TAT, tyrosine aminotransferase; HPPR, hydroxyl phenylpyruvate reductase; PAL, phenylalanine ammonia lyase; C4H, cinnamate 4-hydroxylase; 4CL, hydroxycinnamate-CoA ligase; RAS, rosmarinic acid synthase; CYP, cytochrome P450 enzymes; CHS, chalcone synthase; CHI, chalcone isomerase; FLS, flavonol synthase; F3H, flavanone 3-hydroxylase; F3’H, flavonoid 3’-hydroxylase; F3′5′H, flavonoid 3′,5′-hydroxylase; DFR, dihydroflavonol 4-reductase; ANS, anthocyanin synthase; HCT, hydroxyl cinnamoyl transferase; CCR, cinnamoyl-CoA reductase; C3’ H, coumarate 3’-hydroxylase; COMT, caffeic acid *O*-methyltransferase; CCoAMT, caffeoyl-CoA *O*-methyltransferase; F5H, ferulate 5-hydroxylase; GT, glycosyltransferase; CAD, cinnamyl alcohol dehydrogenase.

To investigate the underlying mechanism of AtPAP1 in *S. miltiorrhiza*, we compared the global expression profiles of AtPAP1-14 versus the WT. Two cDNA libraries were sequenced by Illumina deep sequencing to obtain 3,567,545 and 3,679,557 clean tags for AtPAP1-14 and WT, respectively (Supplementary Table S2). These sequences were mapped to the *S. miltiorrhiza* reference transcriptome database (Hua et al., 2011). Tags with a complete match or a mismatch of only one base pair received further consideration. A comparable proportion of tags (44.24% for PAP1-14 and 50.96% for the control) matched the *S. miltiorrhiza* transcriptome (Supplementary Table S2). Transcriptome analysis identified 4152 differentially expressed genes (DEGs), with 1,601 unigenes being up-regulated and 2551 down-regulated in AtPAP1-14 when compared with expression in the WT (Figure 1A). Our KEGG analysis identified 23 significantly enriched metabolic pathways for those DEGs (Supplementary Table S3). Their functions included photosynthesis, as well as the biosynthesis of plant hormones, terpenoids, steroids, and phenylpropanoids. The pathway for phenylpropanoids enriched the most DEGs, 114, among the pathways involved in biosynthesis of secondary metabolites. Expression of four genes (*PAL2, C4H, 4CL*, and *RAS*) in the phenylpropanoid pathway and the tyrosine pathway gene *TAT1* was increased more than twofold (Supplementary Table S4 and Figure 1B), a finding in accord with our earlier report (Zhang et al., 2010). The DEGs related to secondary metabolites, including those in the shikimate and flavonoid pathways and genes active in terpenoid backbone biosynthesis, are listed in Supplementary Table S4. Among the 10 DEGs in the terpenoid pathway, only one (encoding DXS) was up-regulated, which is consistent with the result from our metabolome analysis that showed no obvious changes in the level of tanshinones due to overexpression.

In addition to analyzing the abundance of genes for enzymes in the biosynthesis pathway for secondary metabolites, we analyzed any TFs for which expression was changed by at least twofold between OE and WT plants. The 42 identified DEGs included those within the bHLH superfamily (8 members), WRKY superfamily (8), myeloblastosis (MYB) superfamily (6), and NAC superfamily (5) (Supplementary Table S5). We then focused on the bHLH genes because that family helps regulate the phenylpropanoid pathway. Among those eight bHLHs, the coding sequence of unigene37696 shows higher amino acid identity with MYC-RP (BAA75513, 79%), MYC-GP (BAA75514, 79%), AmDEL2 (AEM63394, 57%) and AmDEL (AAA32663, 56%), which are known to modulate flavonoids accumulation (Gong et al., 1999; Wang et al., 2016). This indicated that it has a critical role in phenolics biosynthesis.

### Sequence Analysis and Subcellular Localization of SmbHLH51

Using unigene37696 and currently available transcriptome data, we experimentally isolated cDNA from this gene and initially named it *SmMYC* (GenBank Accession Number: KT215166). This gene contains a 1,911-bp open reading frame (ORF) that is predicted to encode a 636-amino acid (aa) protein with a molecular weight (MW) of 69.7 kDa and an isoelectric point (pI) of 5.70. We then used PCR to amplify a 3,420-bp genomic sequence from its genomic DNA. Comparison of the genomic DNA and cDNA sequences revealed that *SmMYC* harbors eight exons. Sequence analysis indicated that SmMYC contains not only a typical bHLH domain (440–489 aa) but also a bHLH-MYC_N pfam (13–196 aa). We compared the gene sequence of *SmMYC* with that of 127 *bHLH*s identified based on the entire genome sequence of *S. miltiorrhiza* (Zhang et al., 2015). The sequence of *SmMYC* is almost completely consistent with that of *SmbHLH51* except that the latter is missing 12 bp, i.e., the ORF length for *SmbHLH51* is 1,899 bp. We speculate that the missing 12 bp is probably an error caused by *de novo* assembly. To keep the consistence of gene name, *SmMYC* was renamed *SmbHLH51* in this study.

**FIGURE 2:**
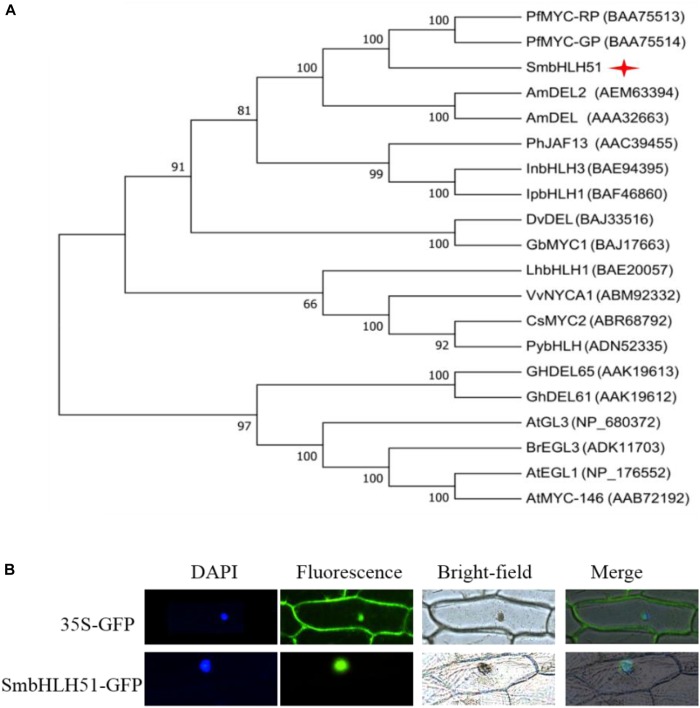
Sequence analysis of *SmbHLH51* and subcellular localization. **(A)** Phylogenetic tree of SmbHLH51 and bHLH protein sequences from other species. **(B)** Subcellular localization of SmbHLH51 in onion epidermal cells.

Alignment of protein sequences between SmbHLH51 and homologous bHLH TFs revealed the presence of a typical bHLH domain and N-terminal conserved domains, including BOX11, BOX18, and BOX13 (Supplementary Figure S1), which interact with R2R3-MYB TFs (Heim et al., 2003; Pires and Dolan, 2010). We then conducted a phylogenetic analysis of bHLH IIIf proteins from various plant species, and found that SmbHLH51 had a close relationship with MYC-RP/MYC-GP from *P. frutescens*, AmDEL and AmDEL2 from *Antirrhinum majus* (Figure 2A). Subcellular localization assay showed that SmbHLH51 was localized to the nucleus (Figure 2B).

### *SmbHLH51* Is Directly Activated by AtPAP1

**FIGURE 3:**
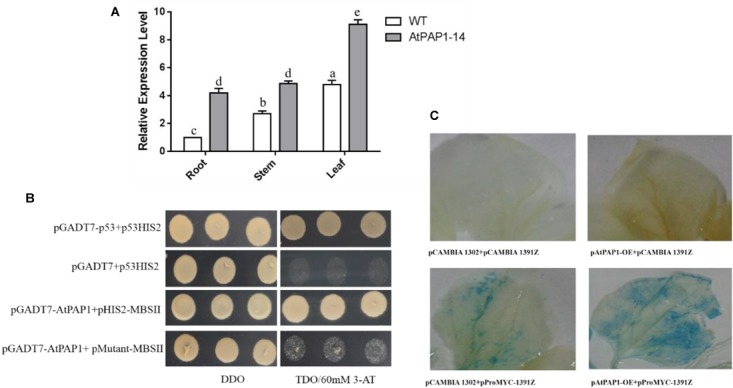
The relationship between AtPAP1 and the *SmbHLH51* gene. **(A)** Relative expression level of *SmbHLH51* in AtPAP1-14 and wild type (WT) roots. The results were analyzed using the comparative Ct method and presented as fold changes compared with the WT. The data represent means ± SD of three independent experiments. One-way ANOVA (followed by a Turkey comparison) tested for significant differences among the means (indicated by different letters at *p* < 0.01). **(B)** Interaction of AtPAP1 with the MBSII element as revealed by yeast one-hybrid assays. AtPAP1 was fused to GAL4 activation domain (AD). The core sequence of *SmbHLH51* promoter MBSII elements were synthesized and cloned into pHIS2 to construct pHIS2-MBSII. Recombinant vectors were co-transformed into yeast strain Y187 and transformed cells were cultured on SD/-Leu/-Trp medium (DDO), then selected on SD/-Leu/-Trp/-His medium (TDO) supplemented with 60 mM 3-amino-1,2,4-triazole (3-AT) to examine protein-DNA interaction. The p53HIS2/pGADT7-p53 and p53HIS2/pGADT7 served as positive control and negative control, respectively. **(C)** AtPAP1 activates *SmbHLH51* promoter via transient expression in tobacco leaves. The effector plasmid pAtPAP1-OE contains AtPAP1 driven by the CaMV 35S promoter. The reporter plasmid pProbHLH51-1391Z contains the GUS reporter gene driven by *SmbHLH51* promoter.

Results from RT-qPCR further validated that the expression level of *SmbHLH51* significantly increased in PAP1-14 when compared with the WT (Figure 3A), which is consistent with our transcriptome data. Analysis of the promoter region indicated that MBSII elements (AAAAGTTAGTTA) were located -1,435 bp upstream of the translation initiation codon ATG, a MYB binding site involved in regulating genes for flavonoid biosynthesis (Supplementary Figure S2). Our Y1H results showed that AtPAP1 could specifically bind to the MBSII elements *in vivo* (Figure 3B).

We isolated the promoter of *SmbHLH51* and used it to examine whether AtPAP1 could directly activate the expression of *SmbHLH51* by *Agrobacterium*-mediated transient expression in tobacco (*Nicotiana benthamiana*). Results from GUS (β-glucuronidase) histochemical staining suggested that, indeed, AtPAP1 directly induced *SmbHLH51* (Figure 3C). Taken together, our results based on RT-qPCR, Y1H and GUS histochemical staining indicated that *SmbHLH51* is directly activated by AtPAP1.

### AtPAP1 Interacts With SmbHLH51

**FIGURE 4:**
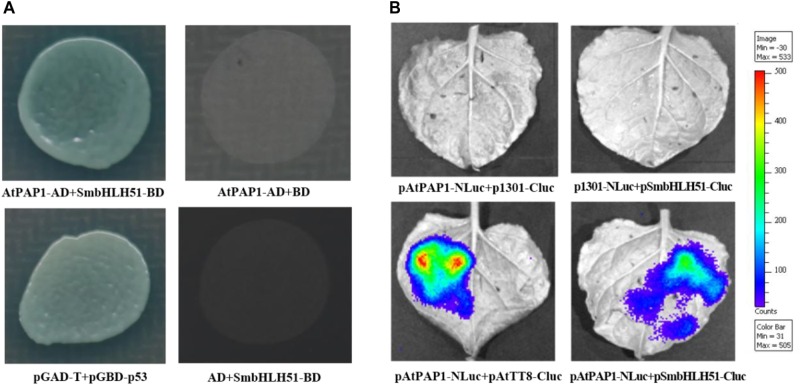
AtPAP1 physically interacts with SmbHLH51. **(A)** Y2H assay to test interactions of AtPAP1 with SmbHLH51. AtPAP1 was fused with activation domain while SmbHLH51 was fused with DNA-binding domain. **(B)** LCI assay to detect interactions of AtPAP1 with SmbHLH51. AtPAP1was fused with N-terminal of luciferase to produce pAtPAP1-NLuc. AtTT8 (GenBank Accession Number NM_117050) and SmbHLH51 were fused with C-terminal of luciferase to produce pAtTT8-Cluc and pSmbHLH51-Cluc, respectively.

The bHLH proteins within the IIIf subgroup (i.e., TT8, GL3, EGL3, and AtMYC1) can interact with various R2R3-MYBs, such as TT2 and PAP1–4 (Xu et al., 2015a). This implies that AtPAP1 has the ability to form a protein complex with IIIf bHLH factor SmbHLH51. Yeast cells co-transformed with vectors harboring AtPAP1-AD/SmbHLH51-BD and the pGBKT7/pGADT7 empty vector could not grow on plates holding a synthetic dextrose/-Ade/-His/-Leu/-Trp medium. However, the yeast cells transformed with AtPAP1-AD and SmbHLH51-BD grew normally, suggesting that AtPAP1 interacts with SmbHLH51 in yeast (Figure 4A).

To confirm those Y2H results, we performed a firefly luciferase complementation imaging (LCI) assay to detect the interaction between AtPAP1 and SmbHLH51. Whereas AtPAP1 was fused with the N-terminal to the NLuc fragment, SmbHLH51 was fused with its C-terminal to the CLuc fragment. As expected, co-injection of the vector constructs into the tobacco leaves presented the same interaction features of the proteins (Figure 4B) that had been observed in the Y2H test. Therefore, all of our Y2H and LCI results demonstrated that AtPAP1 interacts with SmbHLH51 *in vivo*.

### Generation of Transgenic *S. miltiorrhiza* Plants

**FIGURE 5:**
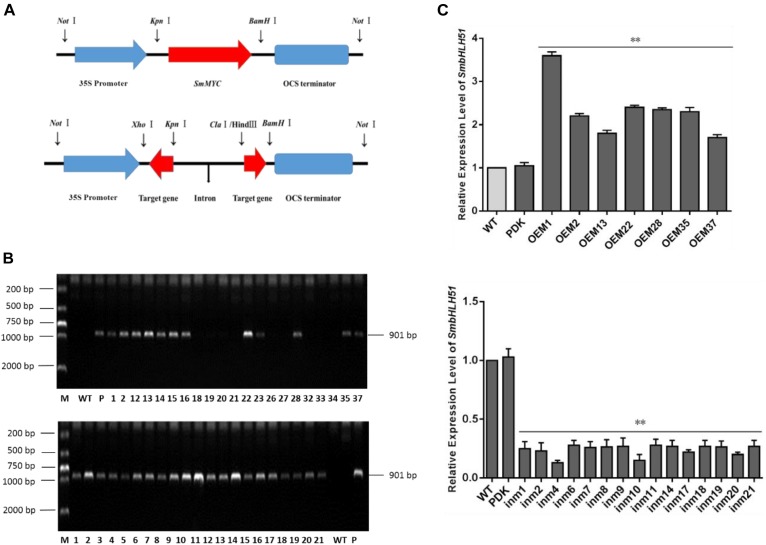
Detection of *SmbHLH51*-overexpressing and *SmbHLH51-RNAi* transgenic lines. **(A)** Sketch map of overexpression and transcription box for RNAi vectors. **(B)** PCR amplification product of 35S promoter from transgenic *Salvia miltiorrhiza* gDNA. Lanes: M, DL2000 DNA marker; P, plasmids as positive control; WT, wild-type plants as negative control. **(C)** Relative expression levels of *SmbHLH51* in *SmbHLH51*-overexpressing transgenic lines (OE) and *SmbHLH51*-RNAi transgenic lines (in). The data represent means ± SD of three independent experiments. Level of *SmbHLH51* in the wild type was set as 1. ^∗∗^*p* < 0.01 compared with the wild type (Student’s *t*-test).

Explants were transformed via *Agrobacterium* mediation with overexpression plasmid pAK-SmbHLH51, RNAi vector pSmbHLH51-RNAi (Figure 5A), and the empty control pAK. After selective culturing on a kanamycin medium, PCR-amplification was conducted with genomic DNA as templates. An expected 901-bp fragment of the CaMV 35S promoter was amplified in the transgenic *S. miltiorrhiza* plants but not in the WT (Figure 5B). Our PCR analysis showed that the positive transformation rate of *SmbHLH51-*overexpressing (*SmbHLH51*-OE) and *SmbHLH51*-RNAi lines was 70% and 95%, respectively. We then conducted RT-qPCR analysis of those transgenics (Figure 5C). From this we selected for further examination two *SmbHLH51*-OE lines (OE1 and OE35) with higher *SmbHLH51* transcription levels and two *SmbHLH51*-RNAi lines (in4 and in10) with lower *SmbHLH51* transcription levels. Our controls were the WT and those plants that had been transformed with the corresponding empty vector (PDK).

### Phenotypes of *SmbHLH51*-Overexpressing and *SmbHLH51*-RNAi *S. miltiorrhiza*

**FIGURE 6:**
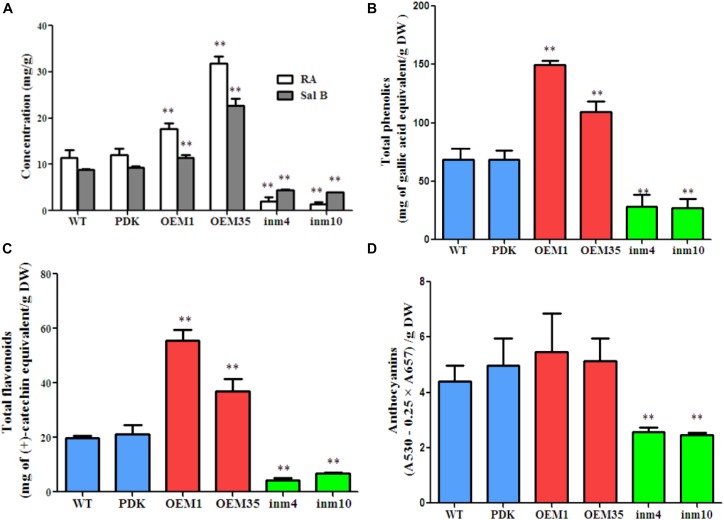
Comparison of secondary metabolite concentrations between transgenic lines and controls. **(A)** Levels of RA and Sal B in root extracts. **(B–D)** Levels of total phenolics **(B)**, total flavonoids **(C)**, and anthocyanins **(D)** from root extracts. The data represent means ± SD of three independent experiments. ^∗∗^*p* < 0.01 compared with the wild type (Student’s *t*-test).

To investigate whether phenolic acids metabolism was modified in our *SmbHLH51*-transgenic plants, we extracted their phenolic acids and separated them via HPLC. The results showed that levels of RA and Sal B did not differ significantly between the WT and PDK. However, when compared with the WT, the concentrations of both RA and Sal B were increased significantly (*P* < 0.01) in OE1 and OE35 (Figure 6A). For example, the amount of RA was 1.54-fold and 2.77-fold higher in OE1 and OE35, respectively, than in the WT, while Sal B concentrations were approximately 1.28-fold and 2.54-fold higher, respectively, than in the WT. In contrast, the levels of RA and Sal B were significantly lower (*P* < 0.01) in the *SmbHLH51*-RNAi lines in4 and in10 (Figure 6A), with those reductions being 17.99 % and 12.32%, respectively, for RA and 50.00% and 45.20%, respectively, for Sal B.

We also performed global assays for phenolics, flavonoids, and anthocyanins because they share an upstream core phenylpropanoid metabolism with Sal B. Total phenolics and total flavonoids accumulated at higher levels in the roots of the two *SmbHLH51*-OE lines than in the WT. Differences were significant and corresponded to 2.19-fold and 1.59-fold increases in total phenolics concentrations for OE1 and OE35, respectively, and 2.81-fold and 1.87-fold increases in total flavonoids (Figures 6B,C). No significant changes in anthocyanin concentrations among genotypes were observed (Figure 6D). In contrast, when compared with the WT, the levels of total phenolics, total flavonoids, and anthocyanins were dramatically lower in both *SmbHLH51*-RNAi lines, with decreases being 41% (in4) and 40% (in10) for total phenolics, 22% (in4) and 34% (in10) for total flavonoids, and 56% (in4) and 54% (in10) for anthocyanins (Figures 6B–D). These results implied that *SmbHLH51* is an important regulator of biosynthesis for phenolic acids and flavonoids (including anthocyanins).

### Effect of SmbHLH51 on the Expression of Genes in the Phenolics Pathway

**FIGURE 7:**
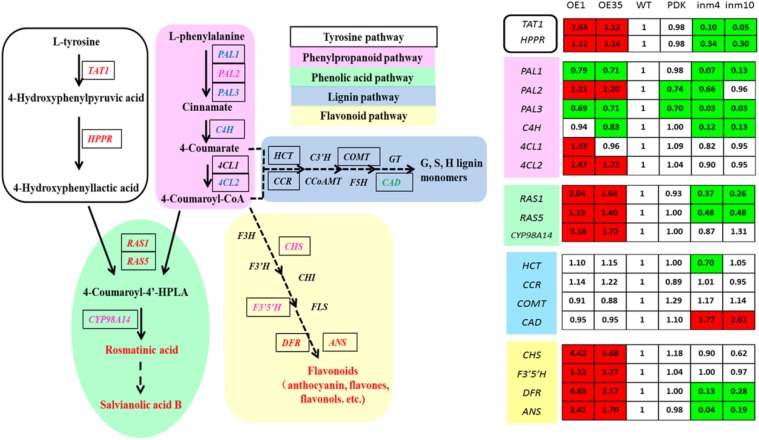
Relative expression levels of 19 phenolics-pathway genes in *SmbHLH51*-overexpressing and *SmbHLH51*-RNAi *Salvia miltiorrhiza* were analyzed by RT-qPCR. The data represent fold changes of phenolics-pathway genes compared with the wild type (WT). Red box indicates the data is significantly upregulated compared with the WT (*p* < 0.05, Student’s *t*-test); green box indicates the data is significantly downregulated compared with the WT (*p* < 0.05, Student’s *t*-test). The following genes were retrieved from GenBank databases: *TAT1* (DQ334606.1), *HPPR* (DQ099741.1), *PAL1* (EF462460.1), *PAL2* (GQ249111.1), *PAL3* (KF220569.1), *C4H* (DQ355979.1), *4CL1* (AY237163.1), *4CL2* (AY237164.1), *RAS1* (FJ906696.1), *RAS5* (KF220573.1), *CYP98A14* (HQ316179.1), *CAD* (HQ162287.1), *CHS* (KF255832.1), *F3’5’H* (MH447665.1), *DFR* (MH447664.1), *ANS* (MH447663.1). *HCT* (Smil-00001226), *CCR* (Smil-00000329) and *COMT* (Smil-00026281) were retrieved from the web portal at http://www.ndctcm.org/shujukujieshao/2015-04-23/27.html.

To evaluate whether these substantial changes in the levels of phenolics in the transgenics were a result of significant alterations in gene expression relative to the control, we examined the transcript levels of 19 genes for enzymes in the phenolics biosynthesis pathway. Relative to expression in the WT, expression in the *SmbHLH51*-OE or *SmbHLH51*-RNAi transgenic plants was markedly different for genes in the core phenylpropanoid pathway and specific branch pathways (Figure 7). Of those 19 genes, 11 were significantly up-regulated in the *SmbHLH51*-OE lines while nine were dramatically down-regulated in the *SmbHLH51*-RNAi lines. Expression of *SmCAD* was unexpectedly increased in the *SmbHLH51*-RNAi lines. Six genes (*SmTAT1, SmHPPR, SmRAS1, SmRAS5, SmDFR*, and *SmANS*) were significantly induced in *SmbHLH51*-OE lines but their expression was dramatically reduced in *SmbHLH51*-RNAi lines. Another five genes (*SmPAL2, Sm4CL2, SmCYP98A14, SmCHS*, and *SmF3’5’H*) were significantly up-regulated in *SmbHLH51*-OE lines but their expression was not significantly changed in *SmbHLH51*-RNAi lines. Three genes (*SmPAL1, SmPAL3*, and *SmC4H*) were markedly down-regulated in *SmbHLH51*-RNAi lines but not significantly altered in *SmbHLH51*-OE lines. Finally, the expression of four genes – *Sm4CL1, SmHCT, SmCCR*, and *SmCOMT* – was not influenced significantly in either *SmbHLH51*-OE or *SmbHLH51*-RNAi lines. All of these findings demonstrated that SmbHLH51 positively regulates many genes that encode enzymes in the pathways for phenolic acid and flavonoid biosynthesis.

The bHLH TFs can form homodimers or heterodimers to bind both canonical and non-canonical E-box sites (CANNTG) (Earley et al., 2006). We analyzed the promoter sequences of the 15 phenolics-pathway genes that were most significantly regulated in *SmbHLH51*-OE and *SmbHLH51*-RNAi transgenic lines and found that most, except *PAL2* and *CAD*, contained E/G-box sequences in their promoters (Supplementary Table S6). We also determined that the promoter regions of 10 genes (*TAT1, HPPR, PAL3, C4H, 4CL2, RAS5, CYP98A14, CHS, DFR*, and *ANS*) contained not only E-box sites but also MBS elements. Whether these genes are directly activated by SmbHLH51 or AtPAP1-SmbHLH51 complex requires further study.

## Discussion

### Global Changes in Transcriptome Caused by Ectopic AtPAP1 Expression in *Salvia miltiorrhiza*

Altering the expression of TFs can be an effective means for coordinately modulating entire metabolic pathways in plants (Massari and Murre, 2000). AtPAP1 is a well studied TF which could be used to stimulate not only anthocyanin accumulation but also the biosynthesis of some valuable phenolic compounds in medicinal plants. For instance, Overexpression of *AtPAP1* enhances chlorogenic acid production in hairy roots of *Platycodon grandiflorum* (Tuan et al., 2014). We previously reported that overexpression of *AtPAP1* in *S. miltiorrhiza* significantly enhanced the levels of not only Sal B, but also total flavonoids, anthocyanins, and lignin (Zhang et al., 2010). However, the responsible mechanism has not been well understood. To improve our knowledge about the regulatory mechanisms by which AtPAP1 promotes phenolic acid biosynthesis, we used transcriptomic analyses to characterize transgenic *S. miltiorrhiza* plants that over-expressed that gene.

In this study, we compared the transcriptomes between *AtPAP1-*overexpressing *S. miltiorrhiza* and the WT. Transcriptome analysis demonstrated that a novel bHLH TF, SmbHLH51, is activated by AtPAP1 in *S. miltiorrhiza*. Usually the bHLH genes are positively regulated by R2R3-MYB TF (Baudry et al., 2006; Bai et al., 2011; Montefiori et al., 2015). Based on previous reports, we propose that bHLHs, which is activated by AtPAP1 and can interact with AtPAP1 in *S. miltiorrhiza*, is potentially involved in the regulation of phenolic acids biosynthesis. The results from our RT-qPCR, Y1H and *Agrobacterium*-mediated transient expression in tobacco indicated that AtPAP1 positively regulates *SmbHLH51* by directly binding to the MBSII element of promoter region (Figure 3). Therefore, the latter gene is functionally identified as a positive regulator of phenolic acid biosynthesis. In addition to its direct transcriptional regulation activities, AtPAP1 interacts with SmbHLH51 and functions as a protein complex to promote the accumulation of phenolics.

### The Integrated Strategy Reveals That SmbHLH51 Is a Putative Regulator of Phenolics Accumulations in *S. miltiorrhiza*

We employed this integrated approach to identify regulatory factors that influence phenolics accumulations because that process has been poorly understood in *S. miltiorrhiza*. The bHLH proteins, comprising one of the largest TF families, regulate various physiological or morphological events, including those in different branches of the flavonoid pathway (Hichri et al., 2011). The bHLHs Booster1 (B) and Red1 (R), which control the anthocyanin biosynthesis pathway in seeds, were first found in *Zea mays* (Chandler and Turks, 1989). Since then, several other bHLH proteins regulating the flavonoid pathway have been identified, including Delila (DEL) in *Antirrhinum majus* (Martin et al., 1991); MYC-RP in *P. frutescens* (Gong et al., 1999); AN1 and JAF13 in *Petunia hybrid* (Quattrocchio et al., 1998; Spelt et al., 2000). AtMYC-146, GLABRA3 (GL3), and TT8 in *Arabidopsis* (Nesi et al., 2000; Ramsay et al., 2003); and VvMYC1 and VvMYCA1 in *Vitis vinifera* (Hichri et al., 2010). Usually the bHLH genes are positively regulated by R2R3-MYB TF (Baudry et al., 2006; Bai et al., 2011; Montefiori et al., 2015). Based on these reports, we propose that bHLHs, which is activated by AtPAP1 and can interact with AtPAP1 in *S. miltiorrhiza*, is potentially involved in the regulation of phenolic acids biosynthesis.

To date, at least 43 bHLH TFs have been identified as regulators of secondary metabolism in at least 21 plant species, with those active components including flavonoids, alkaloids, and terpenoids (Zhang et al., 2015). The bHLHs have been divided into 26 subgroups (Pires and Dolan, 2010), those related to flavonoids are in subgroup IIIf (Montefiori et al., 2015). We also were able to assign SmbHLH51 to the IIIf subfamily based on the bHLH group nomenclature presented above. Members of the same plant bHLH subfamily are frequently involved in the same biological process.

SmbHLH51 shows high similarity with MYC-RP from *P. frutescens* (Figure 2C), which regulates anthocyanin biosynthesis by controlling the transcription of structural genes *PAL, DFR*, and *ANS* in that pathway (Gong et al., 1999). We found here that SmbHLH51 positively regulates not only *DFR, ANS, CHS*, and *F3’5’H* in the flavonoid biosynthesis pathway, but also *PAL* and *C4H* in the core phenylpropanoid pathway, *TAT* and *HPPR* in the tyrosine pathway, and *RAS1 and RAS5* in the phenolic acid pathway (Figure 6). This might explain the significant changes in phenylpropanoid-derived end products in our transgenic lines. We also showed that SmbHLH51 positively regulates different branches of the phenylpropanoid pathway, altering the accumulations of phenolic acids and flavonoids, which is consistent with the function of AtPAP1 in *S. miltiorrhiza*.

### Interactions Between AtPAP1 and SmbHLH51

Although MYB and bHLH can individually regulate phenylpropanoid metabolism and enhance the production of secondary metabolites, many studies have confirmed that they could play more prominent roles when in the form of a MYB/bHLH/WD40 (MBW) protein complex (Gonzalez et al., 2008; Qi et al., 2011; Xu et al., 2015a). The interactions between R2R3-MYB TFs and the bHLHs from subgroup IIIf are among the best-described examples of cooperation between plant transcription regulators (Xu et al., 2015a). In *Arabidopsis*, AtPAP1 can interact with TT8, GL3, EGL3, or AtMYC1, together with TTG1 (WDR family), to form MBW complexes and activate flavonoid biosynthesis (Feller et al., 2011). Our Y2H and LCI results showed that AtPAP1 interacts with SmbHLH51 (Figure 3), indicating that AtPAP1 elicits strong induction of phenolic acids in *S. miltiorrhiza* through an efficient interaction with the endogenous bHLH factor SmbHLH51, so that they are functionally formed as a regulatory transcription complex.

AtPAP1 has a critical role in controlling the production of soluble phenolic compounds in *S. miltiorrhiza* (Zhang et al., 2010) By now only a few endogenous R2R3-MYBs in *S. miltiorrhiza* have been functionally characterized through genetic screening, including *SmMYB39* and *SmMYB36*, two repressors of the phenolic acids pathway (Zhang et al., 2013; Ding et al., 2017). Although SmPAP1, an endogenous R2R3-MYB TF from *S. miltiorrhiza*, was reported to be a positive regulator for the phenolic acid biosynthetic pathway (Hao et al., 2016), its ability in controlling the production of phenolic acids is far from that of AtPAP1 (Zhang et al., 2010). Therefore, it would be very beneficial to identify more endogenous TFs related to phenolic acid biosynthesis in *S. miltiorrhiza*. In the present study, SmbHLH51 was identified as a positive regulator for phenolic acid biosynthesis. We speculate that the endogenous R2R3-MYB, interacting with SmbHLH51, has the same role.

## Conclusion

We have characterized *AtPAP1*-overexpressing plants of *S. miltiorrhiza* through transcriptomic and metabolomic analyses. Our data provide a molecular phenotype for these transgenics in which AtPAP1 strongly promotes the accumulation of phenolics, consistent with the upregulation of genes in the pathway for phenylpropanoid biosynthesis. Through integrative analyses of the metabolome and transcriptome of OE plants, we discovered *SmbHLH51*, a novel bHLH gene that we functionally identified as a positive transcriptional regulator of the phenolics pathway. Our findings demonstrate that AtPAP1 stimulates the production of phenolic acids in *S. miltiorrhiza* by activating the expression of *SmbHLH51* and forming a transcriptional complex with SmbHLH51. This improves our knowledge about the regulatory mechanism of AtPAP1. Further experiments are needed to elucidate the precise molecular mechanism by which AtPAP1/SmbHLH51 regulates the biosynthesis of phenolic acids in *S. miltiorrhiza*.

## Author Contributions

ZW and XC designed and coordinated the study. YW, YZ, LL, XG and BW performed the experiments and analyzed the data. YZ and XC wrote the manuscript.

## Conflict of Interest Statement

The authors declare that the research was conducted in the absence of any commercial or financial relationships that could be construed as a potential conflict of interest.

## Footnotes

1 http://www.genome.jp/kegg/

2 http://gsds.cbi.pku.edu.cn

3 www.ncbi.nlm.nih.gov/BLAST/

4 http://www.expasy.ch/tools/pi_tool.html
