# Supplementary material for: AtPAP1 Interacts With and Activates SmbHLH51, a Positive Regulator to Phenolic Acids Biosynthesis in Salvia miltiorrhiza
Source: Front Plant Sci. 2018 Nov 20;9:1687. doi: 10.3389/fpls.2018.01687 (PMC6255977; doi:10.3389/fpls.2018.01687)
Supplement: Supplementary file 1 [file Table_1.docx]

## *Frontiers in Plant Sciences* Supporting Information

Article title: AtPAP1 Interacts With and Activates SmbHLH51, a Positive Regulator to Phenolic Acids Biosynthesis in *Salvia miltiorrhiza*

Authors: Yucui Wu, Yuan Zhang, Lin Li, Xiaorong Guo, Bin Wang, Xiaoyan Cao, Zhezhi Wang

**Fig. S1** Multiple alignment of SmbHLH51 and homologous bHLHs. The boxed sequences are the potential functional motifs.


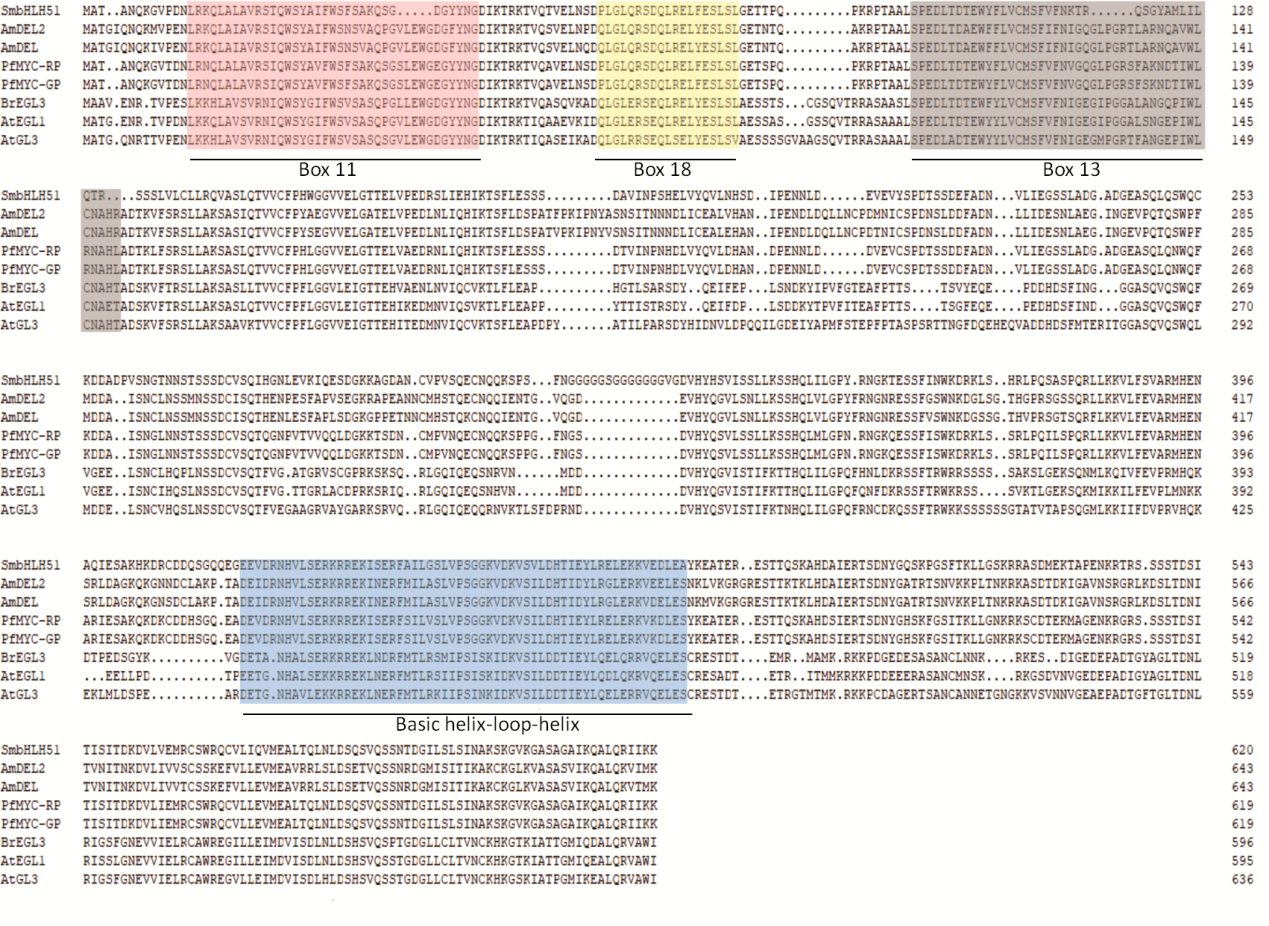


**Fig. S2** Promoter region of the *SmbHLH51*


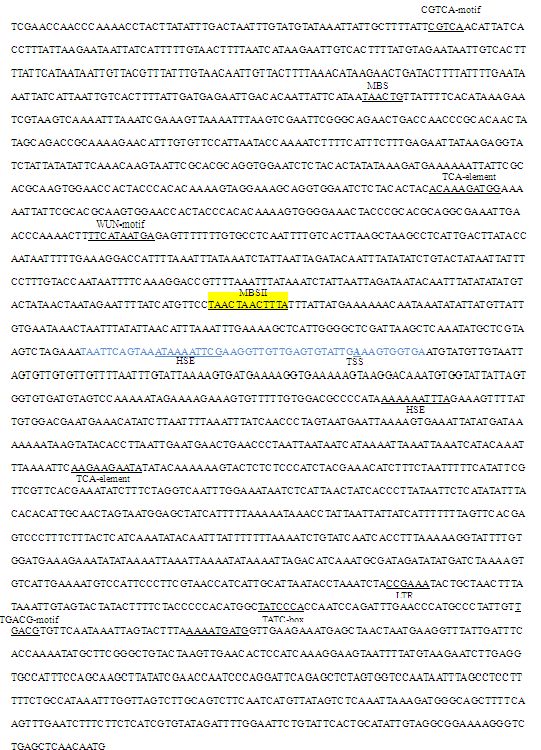


**Table S1** Primers used in experimental procedures

| *Primer* | *Sequence* | |
| --- | --- | --- |
| **Primers used for real-time quantitative PCR** | | |
| SmActin-A | | 5’-GTGGGGCGCCCAGGCACCT-3’ |
| SmActin-S | | 5’-CTTCCTTAATGTCACGCACGATTG-3’ |
| SmbHLH51-A | | 5’-CCGCAAATGACGTCAAGAATTACTG-3’ |
| SmbHLH51-S | | 5’-GGAATGGGCGTAATGTCTCTCTTTT-3’ |
| SmDFR-A | | 5’-CTCACTACTCCATCATAAAGCAAGG-3’ |
| SmDFR-S | | 5’-AGTAGAAAACGGCAGCAATCCT-3’ |
| SmANS-A | | 5’-ACCTTCATCCTCCACAACAT-3’ |
| SmANS-S | | 5’-ACCTTCTCCTTGTTGACCAG-3’ |
| SmRAS1-A | | 5’- GCAAACGAGCACCACCTATCC-3’ |
| SmRAS1-S | | 5’- GTCTTGGAGCGGGGTTTCG-3’ |
| SmRAS5-A | | 5’- GGGAGTGTTGTTCGTGGAGG-3’ |
| SmRAS5-S | | 5’-GCAAAAGTGGGAAGGTGGAA-3’ |
| SmHCT-A | | 5’- GGCCTACCCGAAGACCAAA-3’ |
| SmHCT-S | | 5’- ATGGGCGTGGCTGTGAA-3’ |
| SmCHS-A | | 5’-CGCGATTATGCTTGAGGTTGA-3’ |
| SmCHS-S | | 5’-CACTACTTGATGTCCCATTTCTTGAC-3’ |
| SmF3’5’H-A | | 5’-CATCTACTCCAACATCGGACAGC-3’ |
| SmF3’5’H-S | | 5’-CCCCACATAAGGTTCATCAACAG-3’ |
| SmHPPR-A | | 5’-TGACTCCAGAAACAACCCACATT-3’ |
| SmHPPR-S | | 5’-CCCAGACGACCCTCCACAAG-3’ |
| SmTAT-A | | 5’-CAACTGCTGGTCTTCCACAAAC-3’ |
| SmTAT-S | | 5’-GCGAGCCAAAACGGACA-3’ |
| SmPAL1-A | | 5’-GATAGCGGAGTGCAGGTCGTAC-3’ |
| SmPAL1-S | | 5’-CGAACTAGCAGATTGGCAGAGG-3’ |
| SmPAL2-A | | 5’-GGCGGCGATTGAGAGCAGGA-3’ |
| SmPAL2-S | | 5’-ATCAGCAGATAGGAAGAGGAGCACC-3’ |
| SmPAL3-A | | 5’-CCCGCGATCGGGAACAGGATCAA-3’ |
| SmPAL3-S | | 5’-GCGGCTCTCCATTCCACGATTCA-3’ |
| Sm4CL1-A | | 5’-ATTCGCATTCGCATTTCTCGG-3’ |
| Sm4CL1-S | | 5’-GCGGCGTAGTGCTTCACCTTT-3’ |
| Sm4CL2-A | | 5’- TCGCCAAATACGACCTTTCC-3’ |
| Sm4CL2-S | | 5’-TGCTTCAGTCATCCCATACCC-3’ |
| SmC4H-A | | 5’-CCAGGAGTCCAAATAA CAGAGCCG-3’ |
| SmC4H-S | | 5’-GCCACCAAGCGTTCACCAAG AT-3’ |
| SmCCR-A | | 5’-CTGATGTTGCTTCGCCTTCT-3’ |
| SmCCR-S | | 5’-CATACGTGCCTTCCCCTTG-3’ |
| SmCOMT-A | | 5’-GCCACTAAGAATGTTGTCC-3’ |
| SmCOMT-S | | 5’-TCTGTCCTTTCCTTACCA-3’ |
| SmCAD-A | | 5’-CCCCAA AATCATCCCCACTT-3’ |
| SmCAD-S | | 5’-TCATGGGGCTGTAGGTGGTG-3’ |
| SmCYP98A14-A | | 5’-ACGTGCGTGTTGCTACGAGAC-3’ |
| SmCYP98A14-S | | 5’-CGTCGCCAGTGCTGCAACTAA-3’ |
| **Primers used for vector construction** | | |
| Pro-SmbHLH51-F | | AACTGCAGAACTGACCAACCCGCACAAC |
| Pro-SmbHLH51-R | | CGCGGATCCATGATGGTTACGAAGGGAATGGA |
| 207-SmbHLH51-F | | GGGGACAAGTTTGTACAAAAAAGCAGGCTTCATGGCTACTGCAAATCAA |
| 207-SmbHLH51-R | | GGGGACCACTTTGTACAAGAAAGCTGGGTCAATCTTTTTGATAATTCTCTGAAGAGC |
| 207-AtPAP1-F | | GGGGACAAGTTTGTACAAAAAAGCAGGCTTCATGGAGGGTTCGTCCAAAGG |
| 207-AtPAP1-R | | GGGGACCACTTTGTACAAGAAAGCTGGGTCATCAAATTTCACAGTCTCTCCATCG |
| AtPAP1-NLF | | GGGGTACCATGGAGGGTTCGTCCAAAGG |
| AtPAP1-NLR | | ACGCGTCGACATCAAATTTCACAGTCTCTCCATCG |
| AtTT8-CLF | | GGGGTACCATGGATGAATCAAGTATTATTCCG |
| AtTT8-CLR | | ACGCGTCGACTTGGCATCAATAAAGTTAGGGTC |
| SmbHLH51-CLF | | CGGGGTACCATGGCTACTGCAAATCAAAAGG |
| SmbHLH51-CLR | | CGCGGATCCAATCTTTTTGATAATTCTCTGAAGAGC |
| OEbHLH51-F | | CGGGGTACCTGAGCTTAACAATGGCTACTGC |
| OEbHLH51-R | | CGCGGATCCAAATTGCCAGCATGATCTTC |
| ibHLH51-F | | AAGCTTGGTACCTGTTCCAGATAATCTCAGAAAGCAG |
| ibHLH51-R | | GGATCCCTCGAGTCTTAGGTTGTGGGGTGGTCTC |
| 35S-F | | 5’-TACAAAGGCGGCAACAAACG-3’ |
| 35S-R | | 5’-GCAATGGAATCCGAGGAGGT-3’ |

**Table S2** Categorization and abundance of tags

| *Summary* |  | *Control* | *PAP1-14* |
| --- | --- | --- | --- |
| Raw tag | Total | 3570000 | 3832500 |
|  | Distinct tag | 127842 | 164175 |
| Clean tag | Total number | 3567545 | 3829838 |
|  | Distinct tag number | 126251 | 162367 |
| All tag mapping to gene | Total number | 1817907 | 1694186 |
|  | Total % of clean tag | 50.96% | 44.24% |
|  | Distinct Tag number | 56518 | 58931 |
|  | Distinct Tag % of clean tag | 44.77% | 36.29% |
| Unique tag mapping to gene | Total number | 1793251 | 1645059 |
|  | Total % of clean tag | 50.27% | 42.95% |
|  | Distinct Tag number | 55862 | 58151 |
|  | Distinct Tag % of clean tag | 44.25% | 35.81% |
| All tag-mapped genes | number | 20796 | 21192 |
|  | % of ref genes | 23.03% | 23.47% |
| Unambiguous Tag-mapped Genes | number | 20435 | 20796 |
|  | % of ref genes | 22.63% | 23.03% |
| Unknown tag | Total number | 1726156 | 2088685 |
|  | Total % of clean tag | 48.38% | 54.54% |
|  | Distinct Tag number | 68607 | 100080 |
|  | Distinct Tag % of clean tag | 54.34% | 61.64% |

Clean tags are tags after filtering dirty tags (low quality tags) from raw data;

Distinct tags are different kinds of tags;

Unique tags are the reminder clean tags after removing tags mapped to reference sequences from multiple genes.

**Table S3** List of enriched pathways based on the KEGG database

| *Pathway term* | *Pathway ID* | *DEGs tested* | *P value* | *Q value* |
| --- | --- | --- | --- | --- |
| Ribosome | ko03010 | 95 | 8.80039E-08 | 1.09125E-05 |
| Metabolic pathways | ko01100 | 565 | 6.35599E-07 | 3.94071E-05 |
| Carbon fixation in photosynthetic organisms | ko00710 | 43 | 1.38872E-05 | 0.0006 |
| Porphyrin and chlorophyll metabolism | ko00860 | 24 | 2.18825E-05 | 0.0007 |
| Photosynthesis | ko00195 | 31 | 0.0001 | 0.0011 |
| Photosynthesis - antenna proteins | ko00196 | 14 | 0.0001 | 0.0011 |
| Butanoate metabolism | ko00650 | 27 | 0.0001 | 0.0011 |
| Selenoamino acid metabolism | ko00450 | 21 | 0.0004 | 0.0059 |
| Glutathione metabolism | ko00480 | 32 | 0.0007 | 0.0096 |
| Biosynthesis of plant hormones | ko01070 | 139 | 0.0008 | 0.0096 |
| Sulfur metabolism | ko00920 | 15 | 0.0013 | 0.0142 |
| C5-Branched dibasic acid metabolism | ko00660 | 6 | 0.0017 | 0.0174 |
| Biosynthesis of phenylpropanoids | ko01061 | 114 | 0.0032 | 0.0310 |
| Biosynthesis of terpenoids and steroids | ko01062 | 87 | 0.0036 | 0.0311 |
| Glycine, serine and threonine metabolism | ko00260 | 26 | 0.0038 | 0.0311 |
| Metabolism of xenobiotics by cytochrome P450 | ko00980 | 19 | 0.0053 | 0.0413 |
| Cysteine and methionine metabolism | ko00270 | 43 | 0.0075 | 0.0538 |
| Biosynthesis of alkaloids derived from terpenoid and polyketide | ko01066 | 65 | 0.0087 | 0.0538 |
| Ubiquinone and other terpenoid-quinone biosynthesis | ko00130 | 19 | 0.0088 | 0.0538 |
| Biosynthesis of alkaloids derived from shikimate pathway | ko01063 | 75 | 0.0094 | 0.0538 |
| beta-Alanine metabolism | ko00410 | 18 | 0.0095 | 0.0538 |
| Pyruvate metabolism | ko00620 | 45 | 0.0096 | 0.0538 |
| Nitrogen metabolism | ko00910 | 25 | 0.0101 | 0.0542 |

Pathways with Q value < 0.05 are significantly enriched.

**Table S4** List of the DEGs related to secondary metabolites between AtPAP1-14 and WT

| ***Gene name*** | ***Unigene ID*** | ***Putative function*** | ***P14/WT value*** |
| --- | --- | --- | --- |
| ***Shikimate pathway*** | | | |
| EPSPS | Unigene16477 | 5-enolpyruvylshikimate-3-phosphate synthase [*Dicliptera chinensis*] | 0.43 |
| CS | Unigene52455 | chorismate synthase [*Vitis vinifera*] | 0.13 |
| CM | Unigene4191 | chorismate mutase 1 [*Petunia x hybrida*] | 0.29 |
| Prephenate dehydratase | Unigene25269 | prephenate dehydratase [*Ipomoea trifida*] | 0.03 |
| ***Phenylpropanoid pathway*** | | | |
| PAL2 | Unigene52119 | phenylalanine ammonia-lyase [*Salvia miltiorrhiza*] | 2.79 |
| C4H | Unigene53935 | cinnamate 4-hydroxylase [*Salvia miltiorrhiza*] | 2.07 |
| 4CL2 | Unigene16323 | acyl:coa ligase [*Populus trichocarpa*] | 2.15 |
| RAS | Unigene55331 | Rosmarinic acid synthase [*Salvia miltiorrhiza*] | 2.42 |
| ***Ligin pathway*** | | | |
| HCT1 | Unigene30262 | hydroxycinnamoyl transferase [*Solenostemon scutellarioides*] | 2.58 |
| HCT2 | Unigene53339 | hydroxycinnamoyl-CoA shikimate/quinate hydroxycinnamoyltransferase [*Coffea canephora*] | 0.15 |
| C3H | Unigene56687 | p-coumaroyl shikimate 3'-hydroxylase isoform 2 [*Ocimum basilicum*] | 2.47 |
| COMT1 | Unigene35594 | resveratrol O-methyltransferase [*Vitis vinifera*] | 966.00 |
| COMT2 | Unigene15683 | O-methyltransferase [*Mentha x piperita*] | 3.26 |
| CCoAMT | Unigene39895 | caffeoyl-CoA O-methyltransferase [*Plantago major*] | 0.37 |
| F5H | Unigene16558 | cytochrome P450 [*Verbena x hybrida*] | 0.01 |
| CCR2 | Unigene21918 | cinnamoyl CoA reductase-like protein [*Populus trichocarpa*] | 4.32 |
| CAD | Unigene37246 | cinnamyl alcohol dehydrogenase [*Panax ginseng*] | 0.25 |
| UDP-glucose:sinapic acid glucosyltransferase1 | Unigene44523 | UDP-glycosyltransferase BMGT1 [*Bacopa monnieri*] | 4.15 |
| UDP-glucose:sinapic acid glucosyltransferase2 | Unigene3653 | UDP-glucose:anthocysnin 5-O-glucosyltransferase [*Perilla frutescens var. crispa*] | 0.01 |
| UDP-glucose:sinapic acid glucosyltransferase3 | Unigene52094 | UDP-glucose:glucosyltransferase [*Lycium barbarum*] | 0.01 |
| Peroxidase | Unigene31638 | class III peroxidase [*Phelipanche ramosa*] | 0.32 |
| UDP-glucose coniferyl alcohol glucosyltransferase 1 | Unigene6403 | UDP-glucose:glucosyltransferase [*Lycium barbarum*] | 0.16 |
| UDP-glucose coniferyl alcohol glucosyltransferase 2 | Unigene44523 | UDP-glycosyltransferase BMGT1 [*Bacopa monnieri*] | 4.25 |
| ***Tyrosine pathway*** | | | |
| TAT1 | Unigene38332 | tyrosine aminotransferase [*Salvia miltiorrhiza*] | 2.90 |
| TAT2 | Unigene56449 | tyrosine aminotransferase [*Salvia miltiorrhiza*] | 0.07 |
| ***Flavonoid pathway*** | | | |
| CHI | Unigene5 | chalcone isomerase [*Garcinia mangostana*] | 0.22 |
| F3H1 | Unigene37655 | oxidoreductase [*Capsicum annuum*] | 2.41 |
| F3H2 | Unigene54797 | Os08g0480200 [*Oryza sativa* (japonica cultivar-group)] | 0.04 |
| FLS | Unigene33659 | OSIGBa0101K10.4 [*Oryza sativa* (indica cultivar-group)] | 0.03 |
| F3'H1 | Unigene2612 | cytochrome P450 [*Populus trichocarpa*] | 1.12 |
| F3'H2 | Unigene56687 | p-coumaroyl shikimate 3'-hydroxylase isoform 2 [*Ocimum basilicum*] | 0.26 |
| F3'5'H | Unigene37011 | CYP92B2v1 [*Nicotiana tabacum*] | 1.41 |
| DFR | Unigene51780 | Os03g0818200 [*Oryza sativa* (japonica cultivar-group)] | 0.33 |
| ANR | Unigene55488 | dihydroflavonol 4-reductase [*Solenostemon scutellarioides*] | 0.29 |
| LAR1 | Unigene53731 | phenylcoumaran benzylic ether reductase-like protein Fi1 [*Striga asiatica*] | 6.74 |
| LAR2 | Unigene45625 | TPA: isoflavone reductase-like protein 4 [*Vitis vinifera*] | 0.01 |
| ***Flavone and flavonol pathway*** | | | |
| flavonoid-3-O-  glucosyltransferase | Unigene44523 | UDP-glycosyltransferase BMGT1 [*Bacopa monnieri*] | 4.25 |
| ***Terpenoid backbone biosynthesis*** | | | |
| ACAT | Unigene51402 | acetyl-CoA C-acetyltransferase [*Hevea brasiliensis*] | 0.19 |
| HMGS | Unigene56447 | HMG-CoA synthase [*Nicotiana langsdorffii* x *Nicotiana sanderae*] | 0.41 |
| HMGR1 | Unigene1485 | 3-hydroxy-3-methylglutaryl-coenzyme A reductase [*Salvia miltiorrhiza*] | 0.13 |
| HMGR2 | Unigene53854 | 3-hydroxy-3-methylglutaryl-coenzyme A reductase [*Salvia miltiorrhiza*] | 0.18 |
| DXS | Unigene19248 | 1-deoxy-D-xylulose 5-phosphate synthase 2 [*Salvia miltiorrhiza*] | 2.10 |
| DXR | Unigene31475 | 1-deoxy-D-xylulose-5-phosphate reductoisomerase [*Catharanthus roseus*] | 0.21 |
| CMS | Unigene54786 | 4-diphosphocytidyl-2-C-methyl-D-erythritol synthase [*Stevia rebaudiana*] | 0.15 |
| IDS | Unigene47943 | 4-hydroxy-3-methylbut-2-enyl diphosphate reductase [*Hevea brasiliensis*] | 0.02 |
| IDI | Unigene38060 | isopentenyl diphosphate isomerase [*Ipomoea* sp. Kenyan] | 0.01 |
| GPPS | Unigene41550 | AT4g38460 [*Arabidopsis thaliana*] | 0.20 |

^A^ P14/WT > 1 indicates the gene was up-regulated in the transgenic *Salvia miltiorrhiza* AtPAP1-14, while P14/WT < 1 means the gene was down-regulated as compared to the controls;

P14: transgenic *S. miltiorrhiza* line AtPAP1-14, WT: wild type.

**Table S5** List of transcription factors changed for 2 fold and more between AtPAP1-14 and WT

| ***TF family*** | ***Unigene ID*** | ***Putative function*** | ***P14/WT value*** |
| --- | --- | --- | --- |
| ***MYB*** | Unigene5000 | R2R3 transcription factor MYB108-like protein 1 [*Vitis vinifera*] | 0.08 |
|  | Unigene15501 | transcription factor [*Solanum lycopersicum*] | 0.16 |
|  | Unigene53846 | Myb-like protein [*Nicotiana tabacum*] | 0.44 |
|  | Unigene31858 | MYB-type transcription factor [*Lotus japonicus*] | 0.07 |
|  | Unigene55640 | MYB-CC type transfactor [*Solanum tuberosum*] | 0.23 |
|  | Unigene8745 | MYB transcription factor MYB34 [*Medicago truncatula*] | 0.24 |
| ***bHLH*** | Unigene26759 | bHLH transcription factor PTF1 [*Glycine max*] | 6.60 |
|  | Unigene37696 | bHLH transcription factor MYC-RP [*Perilla frutescens*] | 2.78 |
|  | Unigene23802 | CIB1 (CRYPTOCHROME-INTERACTING BASIC-HELIX-LOOP-HELIX 1); DNA binding / transcription factor [*Arabidopsis thaliana*] | 2.32 |
|  | Unigene28132 | Os08g0506700 [*Oryza sativa* (japonica cultivar-group)] | 2.30 |
|  | Unigene56677 | basic helix-loop-helix (bHLH) family protein [*Arabidopsis thaliana*] | 2.17 |
|  | Unigene54297 | PREDICTED: similar to MYC transcription factor [*Vitis vinifera*] | 0.04 |
|  | Unigene34576 | PREDICTED: hypothetical protein [*Vitis vinifera*] | 0.11 |
|  | Unigene34696 | basic helix-loop-helix protein [*Nicotiana tabacum*] | 0.16 |
| ***NAC*** | Unigene14243 | NAC domain protein, IPR003441 [*Populus trichocarpa*] | 0.02 |
|  | Unigene19221 | nam-like protein 3 [*Petunia x hybrida*] | 0.02 |
|  | Unigene21914 | Os06g0139700 [*Oryza sativa* (japonica cultivar-group)] | 0.10 |
|  | Unigene21129 | transcription factor [*Capsicum annuum*] | 0.10 |
|  | Unigene48809 | NAP-like transcription factor [*Vitis vinifera*] | 0.14 |
| ***WRKY*** | Unigene3806 | TTG2 (TRANSPARENT TESTA GLABRA 2); WRKY44 transcription factor [*Vitis thunbergii*] | 0.02 |
|  | Unigene34170 | WRKY7 [*Glycine max*] | 7.34 |
|  | Unigene26683 | AtIDD4 (*Arabidopsis thaliana* Indeterminate(ID)-Domain 4); transcription factor | 6.36 |
|  | Unigene54910 | SPF1-like DNA-binding protein [*Cucumis sativus*] | 6.29 |
|  | Unigene43912 | photoreceptor-interacting protein-like [*Zea mays*] | 2.71 |
|  | Unigene56047 | transcriptional factor WRKY I [*Boea hygrometrica*] | 2.48 |
|  | Unigene54882 | DNA-binding protein NtWRKY3 [*Nicotiana tabacum*] | 2.11 |
|  | Unigene55308 | WRKY transcription factor 2 [*Solanum tuberosum*] | 0.00 |
| ***C3H*** | Unigene56037 | zinc finger (C3HC4-type RING finger) family protein [*Arabidopsis thaliana*] | 5.95 |
|  | Unigene32694 | zinc finger (C3HC4-type RING finger) family protein [*Arabidopsis thaliana*] | 4.39 |
|  | Unigene218 | Os10g0204100 [*Oryza sativa* (japonica cultivar-group)] | 3.30 |
|  | Unigene36785 | zinc finger (C3HC4-type RING finger) family protein [*Arabidopsis thaliana*] | 2.72 |
|  | Unigene32433 | Putative finger family protein [*Brassica oleracea*] | 2.71 |
|  | Unigene31920 | Zinc finger, RING-type [*Medicago truncatula*] | 0.36 |
| ***Homeobox*** | Unigene38104 | contains similarity to homeobox domains [*Arabidopsis thaliana*] | 0.01 |
|  | Unigene48152 | homeobox gene 13 protein [*Arabidopsis thaliana*] | 0.09 |
|  | Unigene7467 | homeodomain leucine zipper protein [*Solanum lycopersicum*] | 0.33 |
| ***GRAS*** | Unigene37311 | GRAS family transcription factor [*Populus trichocarpa*] | 0.18 |
|  | Unigene36163 | DELLA domain GRAS family transcription factor [*Populus trichocarpa*] | 0.24 |
|  | Unigene7183 | DELLA domain GRAS family transcription factor [*Populus trichocarpa*] | 0.33 |
| ***TCP*** | Unigene26069 | TCP-1 chaperonin-like protein [*Arabidopsis thaliana*] | 0.42 |
| ***RAV*** | Unigene15391 | RAV [*Nicotiana tabacum*] | 3.84 |
| ***VOZ*** | Unigene24343 | VOZ1 (VASCULAR PLANT ONE ZINC FINGER PROTEIN); transcription activator [*Arabidopsis thaliana*] | 3.79 |
| ***SBP*** | Unigene26255 | transcription factor squamosa promoter binding protein-like [*Eucalyptus grandis*] | 0.01 |

^A^ P14/WT > 1 indicates the gene was up-regulated in the transgenic *Salvia miltiorrhiza* AtPAP1-14, while P14/WT < 1 means the gene was down-regulated as compared to the controls;

P14: transgenic *S. miltiorrhiza* line AtPAP1-14, WT: wild type.

**Table S6** E/G box and MBS elements identified in the promoter regions of 15 phenolic pathway genes significantly regulated in *SmbHLH51*-OE and *SmbHLH51*-RNAi transgenic lines

| *Gene* | *E/G-box (position upstream ATG)* | *MYB binding site* |
| --- | --- | --- |
| TAT1 | CACGTT(-728);CACGTG(-231); CACGAC(-632);CACGTC(-641) | CAACTG(-11); AACCTAA(-936) |
| HPPR | CACGAC(-170);CACGTG(-100) | AACCTAA(-223; -341) |
| PAL1 | CACGAC(-862);CACGTT(-198); CACGTC(-631) | No |
| PAL2 | No | No |
| PAL3 | CACGTC(-125);CACATGG(-1133) | AACCTAA(-1320) |
| C4H | CACGTC(-254) | CAACTG(-1436; -1412) |
| 4CL2 | CACGTT(-414;-408);CACGTC(-322) | AACCTAA(-96) |
| RAS1 | CACGAC(-958);TACGTG(-496) | No |
| RAS5 | CACGTC(-128) | CAACTG(-1169) |
| CYP98A14 | CACGTG(-618) | TAACTG(-887); AACCTAA(-490) |
| DFR | CACGTA(-295); CACGTG(-112) | TAACTG(-1411) |
| ANS | CACGTC(-601); TACGTG(-106) | TAACTG(-405) |
| CHS | CACGTA(-328) | CAACTG(-301) |
| F3’5’H | CACGTG (-690);TACGTG(-239) ; CACGTC (-190) | No |
| CAD | No | MBS CAACTG(-279) |
